# An evaluation of the Clarity 3D ultrasound system for prostate localization

**DOI:** 10.1120/jacmp.v13i4.3753

**Published:** 2012-07-05

**Authors:** Don Robinson, Derek Liu, Stephen Steciw, Colin Field, Helene Daly, Elantholi P. Saibishkumar, Gino Fallone, Matthew Parliament, John Amanie

**Affiliations:** ^1^ Department of Physics University of Alberta Edmonton AB; ^2^ Department of Oncology University of Alberta Edmonton AB; ^3^ Department of Medical Physics Cross Cancer Institute Edmonton AB; ^4^ Department of Radiation Oncology Cross Cancer Institute Edmonton AB; ^5^ Department of Radiation Oncology Princess Margaret Hospital Toronto ON Canada

**Keywords:** prostate, 3D ultrasound, image‐guided radiation therapy

## Abstract

The purpose of this study is to evaluate the accuracy and precision of the Clarity 3D ultrasound system to track prostate gland positional variations due to setup error and organ motion. Seventeen patients (n=17) undergoing radical external beam radiation therapy for localized prostate cancer were studied. Subsequent to initial reference ultrasound and planning CT scans, each patient underwent seven repeat weekly tracking CT and ultrasound (US) scans during the course of treatment. Variations in the location of the prostate between reference and tracking scans were measured. Differences reported by CT and ultrasound scans are compared. Ultrasound tracking was initially performed clinically by a group of trained general users. Retrospective prostate localization was then performed by a trained dedicated user upon the original raw data set and also a reduced data set derived from the original by an expert user from Resonant Medical. Correlation accuracy between ultrasound and CT shifts acquired and delineated by a pool of trained general users was deemed unacceptable for radiotherapy purposes. A mean discrepancy between CT and US localizations of greater than 10 mm, with a 5 mm or greater discrepancy rate of nearly 90%, was observed. Retrospective analysis by a dedicated user of both the original and Resonant Medical reduced data sets yielded mean CT‐Us discrepancies of 8.7 mm and 7.4 mm, respectively. Unfortunately, the 5 mm or greater CT‐US discord rate for these retrospective analyses failed to drop below 80%. The greatest disparity between CT and ultrasound was consistently observed in the superior–inferior direction, while greatest agreement was achieved in the lateral dimension. Despite an expert reanalysis of the original data, the Clarity ultrasound system failed to deliver an acceptable level of geometric accuracy required for modern radiotherapy purposes.

PACS numbers: 8755ne, 87.56Da, 87.63dh

## I. INTRODUCTION

Mounting evidence supports improved disease‐free survival with dose‐escalated radiation therapy using both 3D conformal and intensity‐modulated radiation therapy.^(^ ^1^ ^,^ ^2^ ^)^ The use of tighter margins requires more accurate target localization. Patient setup with the use of portal images is associated with significant limitations primarily due to a reliance on bony landmarks rather than the intended soft target tissue.^(^ ^3^ ^)^ Other methods of verification have been explored in order to improve the accuracy of setup while limiting labor and technical costs. The merits and limitations of these approaches are discussed below. This study assesses the accuracy and precision of the Clarity three‐dimensional ultrasound system (Resonant Medical, Montreal, QC) when used as recommended by the manufacturer to correct for positional uncertainties due to setup error and motion of the prostate gland during the course of external beam radiation therapy.

Implantation of metallic gold seeds into the prostate periphery prior to treatment has been used to aid in radiographic localization of the prostate during radiation delivery. Litzenberg et al.^(^ ^4^ ^)^ showed that positional errors in all directions can be reduced significantly using of this method. The disadvantages of this invasive procedure include the expertise and time required, risk of seed migration which may adversely affect accuracy, and prolonged treatment time.

Kilovoltage CT scanning is a verification method utilizing direct visualization of soft tissues with a high degree of accuracy. Lattanzi et al.^(^ ^5^ ^)^ demonstrated a maximal portal placement error of 3 mm using this technique in six patients. Hoogeman et al.^(^ ^6^ ^)^ also showed that systematic errors can be reduced by a factor of two. Both studies required patient transfer from the simulation room to treatment area, which introduces potential errors into the process. Ghilezan et al.^(^ ^7^ ^)^ evaluated the accuracy of online image‐guided IMRT by performing multiple scans with on‐board cone‐beam CT during treatment in 22 prostate cancer patients. The average equivalent uniform target dose was improved, along with significantly improved bladder and rectum sparing. Sequential CT examinations incur not only significant costs in terms of personal and technical resources, but also require the presence of a CT scanner in each treatment room. This limitation has diminished in recent years through the development of on‐board cone‐beam imaging, which is now more readily available.

Ultrasound is a proven modality for prostate localization and has several advantages including relatively low cost, avoidance of invasive seed placement procedures, and the potential of reduced patient setup times. It is also nonionizing. Latanzi et al.^(^ ^8^ ^)^ compared a two‐dimensional ultrasound‐based targeting system (BAT; NOMOS Corporation, Sewickley, PA) with CT scans for verification of boost phase 3D CRT in 10 prostate cancer patients. In their study, the BAT ultrasound system was found to correlate with CT within a small absolute magnitude of difference. Mean differences in isocenter localization between CT and ultrasound of 3 mm, 4.6 mm, and 2.6 mm in the antero–posterior, supero–inferior, and lateral directions were observed. An update of this study with 35 patients revealed a similar correlation. Chandra et al.^(^ ^9^ ^)^ published their experience of the BAT system with 147 prostate cancer patients treated with IMRT and concluded that the quality of the images by the BAT system was acceptable in 95% of cases, and major alignment adjustments by radiation therapists were required only 3% of the time.

Ultrasound‐based verification methods may be subject to interuser variability in target localization. Serago et al.^(^ ^10^ ^)^ evaluated the interuser variability of BAT ultrasound guidance using 38 patients with prostate cancer. Two consecutive independent ultrasound localization sessions were performed per patient, each by an independent operator. Interpair localization differences of < 1 mm were obtained at a frequency of approximately 50% in the anterior–posterior and superior–inferior directions and 80% laterally. Discrepancies of < 3 mm were observed in all directions for between 80% and 90% of all paired scans. A BAT interuser variability study by Fuss et al.^(^ ^11^ ^)^ revealed user experience to be a significant factor in the overall accuracy obtained with this form of ultrasound guidance.

With the goal of reducing interuser variability, procedure duration, and operator‐dependence, a three‐dimensional ultrasound system named Clarity was developed. The Clarity system acquires 3D ultrasound pelvic data with a 2D abdominal ultrasound probe outfitted with positional sensors, which is swept across the patient's region of interest. An infrared camera is used to track these sensors so that the position and orientation of each 2D image may be determined in order to reconstruct a 3D dataset. Using this system, target verification and patient alignment may be completed within 90 seconds. The Clarity system is intended to be used largely independent of CT by radiation technologists with training provided by Resonant Medical. A prospective clinical trial with 40 prostate cancer patients (217 alignment procedures) was conducted by Cury et al.^(^ ^12^ ^)^ comparing the BAT system and the Clarity system. A difference in paired BAT‐ and Clarity‐measured prostate displacements was found to be statistically significant in the lateral and supero–inferior directions. The Clarity system was compared with CT scans which were acquired during the treatment of 10 patients. Clarity was found to correlate with CT with regard to prostate displacement in all directions. The authors concluded that Clarity displacements were consistent with CT displacements and produced greater prostate alignment accuracy as compared to the BAT system. A similar 3D ultrasound system (SonArray, ZMed Inc., Ashland, MA) is under evaluation and initial clinical experience is reported as encouraging.^(^ ^13^ ^,^ ^14^ ^)^ An investigation by Johnston et al.,^(^ ^15^ ^)^ however, found the Clarity system to be inferior to fiducial markers for daily prostate alignment. In addition to use with prostate, the Clarity system has also been investigated in regard to its application to breast radiotherapy.^(^ ^16^ ^,^ ^17^ ^,^ ^18^ ^,^ ^19^ ^,^ ^20^ ^,^ ^21^ ^)^

Motivated by potential advantages of ultrasound localization, the Clarity system was installed within a CT simulation suite. This study was undertaken to access the prostate localization accuracy of the Clarity system in comparison to CT by repeated scans with both modalities using a cohort of prostate cancer patients slated for radiation therapy. A secondary goal was the comparison of results obtained with trained general users, a trained dedicated user, and an ultrasound expert user (supplied by Resonant Medical).

## II. MATERIALS AND METHODS

Use of the Clarity 3D ultrasound system is intended to improve prostate localization for radiotherapy. This system has been approved for sale on the basis that vendor‐provided training is sufficient to allow for clinical use by general users. As a precursor to clinical utilization, an ultrasound quality assurance (QA) scan must be performed with an alignment phantom in the CT suite to ensure proper registration between coordinate systems. This phantom is positioned in accordance with CT alignment lasers, as this provides an inherent registration between ultrasound and CT coordinate systems.

At the time of a patient's CT scan for treatment planning, an initial reference ultrasound is acquired with the patient remaining in position on the CT support couch. Later these two datasets are registered and a planning reference volume (PRV) corresponding to the prostate gland is contoured on the ultrasound images. In its intended normal clinical use, subsequent tracking ultrasound scans are to be performed (with the patient in treatment position) immediately preceding the delivery of each radiotherapy fraction in order to aid in optimum patient positioning. This is achieved through a set of orthogonal shifts applied to the therapy treatment couch prior to radiation delivery, based on the ultrasound tracking scan. These pretreatment tracking ultrasound sessions were not performed for this study. All ultrasound tracking scans were performed in the CT suit.

Seventeen patients who underwent radical radiotherapy for localized prostate cancer were accrued to this study. All patients received standard pretreatment staging investigations where indicated, including CT of the abdomen, pelvic exam, and a bone scan. A strict bowel and bladder preparation was followed: clear liquid diet for 24 hours prior to scanning, and full bladder and empty rectum (via enema) at scan time. Patients were not scanned if poor bladder filling or rectal emptying was observed. Prior to treatment, each patient underwent initial reference ultrasound and planning CT scans. During the course of their treatments, each patient also submitted to seven weekly tracking sessions (one session per week consisting of a CT scan and two independent ultrasound scans). For the purpose of this investigation, all ultrasound tracking scans were performed in the CT suite (rather than a therapy vault) in order to allow for an accurate comparison with concurrent tracking CT scans. Two tracking ultrasound scans were performed per session: the first immediately preceding the tracking CT scan, and the second immediately following. These two tracking ultrasound scans were each performed by a different operator. These general users consisted of a pool of radiation technologists trained by Resonant Medical who carried out tracking as a part of their routine duties in accordance with the manner of use recommended by Resonant Medical. Variations in prostate location between the initial reference and subsequent tracking scans were independently tracked according to both CT and ultrasound. Differences between the tracking of the prostate as reported by each modality are compared. All CT scans (3 mm slice thickness contiguous spiral acquisition) were performed with a Brilliance Big Bore Scanner (Philips Medical Systems, Cleveland, OH). In order to minimize variability, a single radiation oncologist was responsible for the approval of all contours drawn in both CT and ultrasound.

The Clarity system produces 3D images by means of two ceiling‐mounted cameras which track the motion of infrared light emitting diodes mounted in the handle of its ultrasound transducer. Overall system tracking accuracy is reported to be ±1 mm by the manufacturer. As already mentioned, the unit is calibrated with respect to the coordinate system defined by the CT alignment lasers using an alignment phantom supplied by the manufacturer. Throughout this study, QA using this ultrasound phantom was performed on a daily basis to test this calibration prior to the acquisition of patient data. The alignment lasers for the CT scanner used in this investigation were maintained to an accuracy of ±0.5 mm in each of the cardinal directions. A maximum uncertainty of ±1 mm was also adhered to with regard to the positioning of the ultrasound alignment phantom. Patient positioning for both the planning (reference) and tracking scans was performed to ±1 mm.

Movement of the prostate was assessed by measuring shifts (tracking vs. reference) in the centroid of the target volume delineated according to each modality. These centroid shifts were observed both intra‐ and intermodality, producing motion according to CT, movement as determined by ultrasound, and the difference between these two. All contouring (both CT and ultrasound) was performed using software provided by Resonant Medical on a Clarity (V. 2.0.0.401) research workstation.

The entire ultrasound dataset was subjected to two retrospective analyses. The first of these was performed by a single dedicated technologist (being judged most proficient at both CT and ultrasound from amongst the pool of general users, and afforded dedicated time for this re‐analysis) and involved the inspection of every US image and PRV initially drawn. The modification required of these primal PRVs ranged from nil to minimal to extensive to complete redefinition (in some cases). Scans with images which were deemed suboptimal were excluded from PRV delineation. The resulting dedicated user retrospective dataset was analyzed in like manner to the original. A second revisit of the data was performed by Resonant Medical which provided an independent inspection for images with poor prostate definition and suboptimal US‐CT registration. Those tracking scans deemed to suffer from compromised prostate definition or poor US‐CT registration were culled and the resulting diminished Resonant Medical‐reviewed (RMr) US dataset was formed. This diminished RMr dataset utilized the modified PRVs provided by the dedicated user retrospective analysis.

## III. RESULTS

### A. Initial ultrasound dataset

A total of 136 CT and 272 US shifts were obtained from the 17 accrued patients. Differences between US and CT shifts were determined in the anterior–posterior (AP) (anterior = positive), the lateral (Lat) (patient right = positive), and superior–inferior (SI) (superior = positive) directions for each set (two US and one CT) of tracking scans. The variation between each pair (first (US1) and second (US2)) of ultrasound tracking scans was also computed.

**Figure 1:**
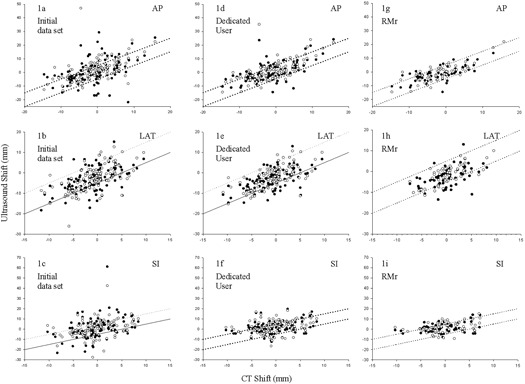
Scatter plots of ultrasound vs. CT shifts (mm) [O CT‐US1, • CT‐US2] in the AP, LAT, and SI directions for the initial dataset (1(a), 1(b), and 1(c), respectively), for the dedicated user dataset (1(d), 1(e), and 1(f), respectively), and for the RMr dataset (1(g), 1(h), and 1(i), respectively). Dotted lines represent CT−US=±5 mm.

**Figure 2:**
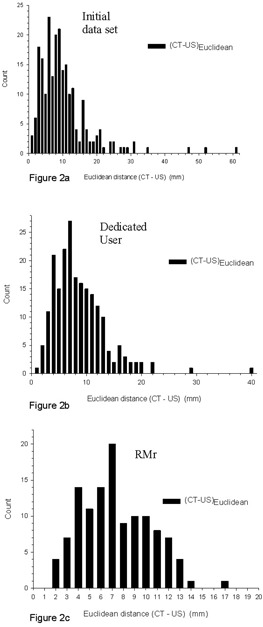
Histograms of the Euclidean distance (mm) between CT and ultrasound shifts for the initial (a), dedicated user (b), and RMr (c) datasets.

Scatter plots of US shifts as a function of CT shifts in the AP, Lat, and SI dimensions are presented in Figs. 1(a), 1(b), and 1(c), respectively. Ideal correlation would manifest as equal shifts recorded by both US and CT at each tracking session, producing linear plots. The data, however, clearly reveals distinct points corresponding to each tracking session which are clustered about the desired US shift =CT shift line. Dotted lines on these graphs represent a ±5 mm deviation between US and CT shifts. The discord between CT and ultrasound tracking is greatest in the AP direction and least in the lateral dimension. A histogram of the Euclidean distance between CT and corresponding ultrasound shifts for this dataset is presented in Fig. 2(a). Here the mean, median, and standard deviation in Euclidean distances between CT and ultrasound shifts are 10.3 mm, 8.7 mm, and ±7.9 mm, respectively. The minimum observed discrepancy between CT and ultrasound‐determined shifts with this dataset is 1.3 mm, while the maximum is 61.4 mm.

**Figure 3:**
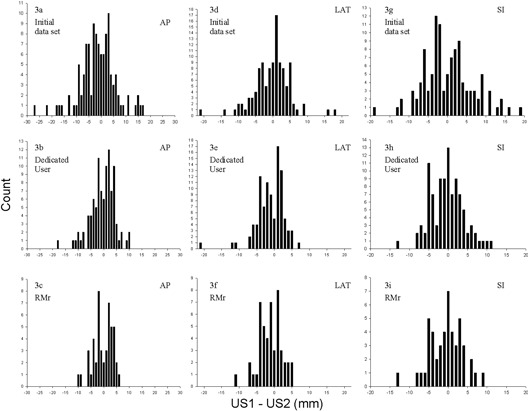
Histograms of the distances between consecutive ultrasound localization determinations (US1–US2) in the AP, LAT, and SI directions for the initial dataset (3(a), 3(d), and 3(g), respectively), for the dedicated user dataset (3(b), 3(e), and 3(h), respectively), and for the RMr dataset (3(c), 3(f), and 3(i), respectively).

**Figure 4:**
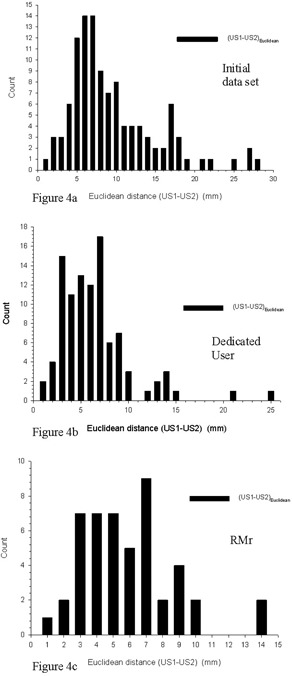
Histograms of the Euclidean distance US1–US2 (mm) between two consecutive ultrasound localization determinations (US1–US2) for the initial (a), dedicated user (b), and RMr (c) datasets.

The preceding graphs reveal not only differences between CT and ultrasound tracking, but also differences between the first and second ultrasound localizations of each tracking session. These ultrasound differences (AP, Lat, and SI) are presented in Figs. 3(a), 3(d), and 3(g). In each cardinal direction (AP, Lat, SI), the results are roughly centered about zero with mean and standard deviation values of −1.2 mm±7.3 mm, −0.3 mm±5.3 mm, and 0.2 mm±6.5 mm, respectively. In its intended clinical use, patient positioning for treatment delivery is to be based not on a statistical mean of numerous scans but rather on a single concurrent ultrasound localization measurement. Thus, a more relevant measure of clinical accuracy is the absolute value of the difference between each pair of ultrasound tracking determinations. When the absolute value of the difference between first and second ultrasound localizations is considered, one has mean and standard deviation values of 5.4 mm±5.0 mm, 3.8 mm±3.7 mm, and 5.1 mm±6.5 mm (AP, Lat, and SI, respectively). Most relevant to clinical patient positioning accuracy is the Euclidean distance between the paired ultrasound localizations, as shown in Fig. 4(a). Here the mean and median distances between first and second ultrasound localization measurements are 9.6 mm and 8.0 mm, with a standard deviation of ±5.6 mm.

### B. Dedicated user retrospective dataset

As a result of the retrospective analysis by a single dedicated technologist, 62 datasets were judged to be of insufficient image quality to allow proper PRV definition and image registration with CT and were excluded from further analysis. Of the 210 US datasets remaining, only 8% of the original PRVs were deemed acceptable without need of modification, 59% were regarded in need of minor modification (< 5 mm change in PRV centroid location), and 33% required major alterations (> 5 mm change in PRV centroid position). These refined PRVs were also used in conjunction with the RMr dataset.

Using this dedicated user dataset, scatter plots of US shifts as a function of CT shifts in the AP, Lat, and SI dimensions are presented in Figs. 1(d)), 1(e), and 1(f), respectively. This data reveal improved conformality to the corresponding CT shifts in comparison to the original general user ultrasound dataset. As before, the dotted lines on these graphs represent a ±5 mm deviation of US shifts from CT shifts. The discord between CT and ultrasound tracking is again greatest in the AP direction and least in the lateral dimension. A histogram of the Euclidean distance between CT and corresponding ultrasound shifts is presented in Fig. 2(b). Here the mean, median, and standard deviation in Euclidean distances between CT and ultrasound shifts are 8.7 mm, 7.6 mm, and ±4.9 mm, respectively. The minimum observed discrepancy between CT and ultrasound determined shifts is 1.0 mm, while the maximum is 40.0 mm.

Differences (AP, Lat, and SI,) between the first and second ultrasound localizations (US1–US2) of each tracking session are presented in Figs. 3(b), 3(e), and 3(h). In each cardinal direction, the results are again roughly centered about zero with mean and standard deviation values of −0.4 mm±4.7 mm, −1.0 mm±4.0 mm, and −0.3 mm±4.2 mm (AP, Lat, and SI, respectively). The absolute value of the difference between first and second ultrasound localizations yields mean and standard deviation values of 3.6 mm±3.0 mm, 3.0 mm±2.8 mm, and 3.3 mm±2.6 mm (AP, Lat, and SI, respectively). The Euclidean distance between the paired ultrasound localizations for this dataset is presented in Fig. 4(b). Here the mean and median Euclidean distance between first and second ultrasound localization measurements are 6.5 mm and 5.9 mm, with a standard deviation of ±3.9 mm.

### C. Resonant Medical‐reviewed (RMr) dataset

A review of the original tracking data by Resonant Medical resulted in 153 of the original 272 scans being judged to be of insufficient image quality to allow proper PRV definition and were excluded from analysis. The refined PRVs from the dedicated user dataset were then adopted for the 119 remaining US tracking scans which form this RMr dataset. Of these 119 RMr scans, only 12% had PRVs which were unaltered from their original definition, 61% were of the minor modification (< 5 mm change in PRV centroid location) class, and 27% belonged to the major alteration group (> 5 mm change in PRV centroid location).

Using this RMr dataset, scatter plots of US shifts as a function of CT shifts in the AP, Lat, and SI dimensions are presented in Figs. 1(g), 1(h), and 1(i), respectively. This data produced further improvement in conformity to the corresponding CT tracking shifts. As before, the dotted lines on these graphs represent a ±5 mm deviation of US shifts from CT shifts. As with the previous two datasets, the discordance between CT and ultrasound tracking is greatest in the AP direction and least in the lateral dimension. A histogram of the Euclidean distance between CT and corresponding ultrasound shifts is presented in Fig. 2(c). Here the mean, median, and standard deviation in Euclidean distances between CT and ultrasound shifts are 7.4 mm, 7.3 mm, and ±3.1 mm, respectively. The minimum observed discrepancy between CT and ultrasound determined shift is 1.8 mm, while the maximum is 17.1 mm.

Differences (AP, Lat, and SI) between the first and second ultrasound localizations of each tracking session for this expert user dataset are presented in Figs. 3(c), 3(f), and 3(i). In each cardinal direction, the results are, as before, roughly centered about zero with mean and standard deviation values of −0.4 mm±3.7 mm, −1.1 mm±3.3 mm, and −0.5 mm±4.2 mm (AP, Lat, and SI, respectively). The absolute value of the difference between first and second ultrasound localizations yields mean and standard deviation values of 3.1 mm±2.0 mm, 2.8 mm±2.0 mm, and 3.3 mm±2.6 mm (AP, Lat, and SI, respectively). The Euclidean distance between the paired ultrasound localizations is presented in Fig. 4(c). Here the mean and median Euclidean distance between first and second ultrasound localization measurements are 6.5 mm and 5.9 mm, with a standard deviation of ±3.9 mm.

## IV. DISCUSSION

The initial dataset was generated by a pool of trained general users for whom both CT and ultrasound scans were but part of their daily radiotherapy related tasks, and was in keeping with the original intended use of the Clarity system. When used in this manner, considerable differences are observed between the two modalities and also between consecutive ultrasound scans. The mean Euclidean distance between CT and ultrasound tracking was 10.3 mm (SD=7.9 mm). Discrepancies between CT and ultrasound of 15 mm or more occurred in 17.9% of all tracking sessions, and 20 mm or more in 9.4% of all tracking scans. Two tracking sessions produced differences of greater than 50 mm, and one produced a difference of greater than 60 mm. Clearly, when used in this manner, this ultrasound system proves a poor substitute for CT tracking. A portion of the observed discrepancy between CT and ultrasound tracking undoubtedly stems from the significant variabilities encountered between consecutive ultrasound scans. With a mean distance between first and second ultrasound determined PRV centroids of 9.6 mm (SD=5.6 mm), differences of 15 mm or more are seen in 17.9% of all tracking sessions and 20 mm or more in 5.4% of all tracking scans.

Retrospective analysis by a single dedicated user resulted in the culling of approximately 23% of the original ultrasound tracking sessions due to an assessment of substandard image quality. Re‐analysis of the remaining tracking scans (including PRV redefinition, where needed) reduced the mean discrepancy between CT and ultrasound shifts to 8.7 mm (SD=4.9 mm). Compared to the original general user dataset, the number of tracking scans where CT and ultrasound shift differences were 15 mm or more was reduced to 9.5% and to 2.9% for deviations of 20 mm or more. Mean discrepancies between consecutive ultrasound tracking scans for this culled dataset were reduced to 6.5 mm (SD=3.9 mm). The occurrence of differences of 15 mm or more was reduced to 3% and deviations of 20 mm or more were diminished to 2%. No differences of greater than 25 mm were observed. In comparison to the results of the original analysis, this retrospective analysis reduced the mean difference between consecutive ultrasound localizations by more than 32% (6.5 mm vs. 9.6 mm), while the average discrepancy between CT and ultrasound was diminished by only 15.5% (8.7 mm vs. 10.3 mm). That the reduction in intra‐ultrasound variability is more than twice the corresponding decrease seen between CT and ultrasound localization is significant. This result strongly indicates that the differences observed between CT and ultrasound localization cannot be explained solely on the basis of the variations experienced with ultrasound. Strict adherence throughout the course of this study to maintaining CT and ultrasound registration to within ±1 mm also mitigates against poor spatial alignment as a root cause.

**Figure 5:**
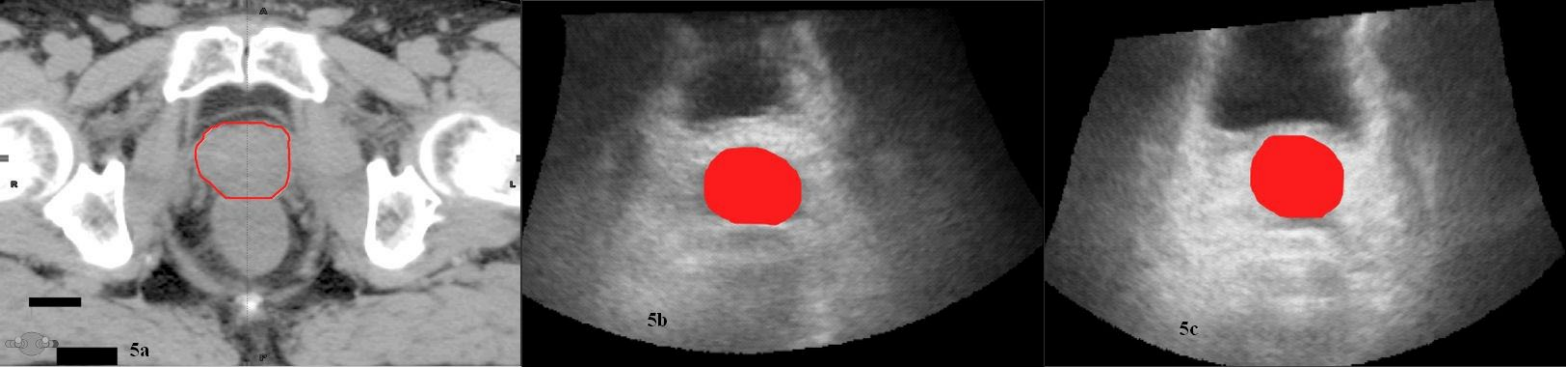
Representative images drawn from the RMr dataset showing the prostate localization provided by a tracking CT scan (a) and its two associated ultrasound tracking scans: US1 (b) and US2 (c).

The retrospective analysis by Resonant Medical resulted in the culling of more than 56% of the original ultrasound tracking sessions owing to a judgment of substandard image quality. A representative set of tracking scans (one CT and its associated first and second ultrasound scans) from this RMr data set is shown in Fig. 5. The differences in prostate definition are clearly evident between all three images. Analysis of the remaining tracking scans reduced the mean discrepancy between CT and ultrasound shifts to 7.4 mm (SD=3.1 mm). The number of tracking scans where CT and ultrasound shift differences were 15 mm or more was reduced to 0.9% with no deviations greater than 17.1 mm. Mean discrepancies between consecutive ultrasound tracking scans for this culled dataset were reduced to 6.0 mm (SD=2.8 mm). The occurrence of intra‐ultrasound differences of 15 mm or more was reduced to nil. In comparison to the results of the original general user analysis, this retrospective analysis reduced the mean difference between consecutive ultrasound localizations by 37.5% (6.0 mm vs. 9.6 mm), while the average discrepancy between CT and ultrasound was diminished by 28.2% (7.4 mm vs. 10.3 mm). Clearly the massive rejection of over 56% of original ultrasound scans on the basis of inferior image clarity or poor registration failed to produce a commensurate increase in agreement between either intra‐ultrasound localizations or CT and ultrasound shifts. The disparity between the reductions achieved for consecutive ultrasound localizations and those between CT and ultrasound is less dramatic than occurred with the dedicated user dataset. The reduction in intra‐ultrasound variability is, however, still significantly greater than the corresponding decrease seen between CT and ultrasound localization and bolsters the conviction that the discrepancies observed between CT and ultrasound localization cannot be explained on the basis of the variations experienced with ultrasound alone. These results suggest, but do not confirm, the existence of a more fundamental discord (at least for the Clarity system) between CT and ultrasound localizations. These results are in contrast to those of Cury et al.^(^ ^12^ ^)^ who found consistency between CT and Resonant Medical ultrasound prostate positional determinations. Both studies (ours and that of Cury) were conducted with ultrasound and CT located in the same room and acquisitions by each modality in immediate temporal proximity. The level of user expertise in the Cury study was not specified. Results presented here reveal a progressive increase in agreement between ultrasound and CT localizations concomitant with expertise in postscan analysis. This suggests that improved results might have been realized if ultrasound scans were performed by dedicated expert users rather than general practitioners. Unfortunately, this was beyond the scope of our investigations and remains an untested hypothesis.

The distribution of discrepancies which are observed with the general user, dedicated user, and expert user analyses between CT and ultrasound and first and second ultrasound tracking scans are summarized in Tables 1 and 2.

**Table 1:** Frequency (%) at which discrepancies between CT and ultrasound localization are greater than, or equal to, specific Euclidean distances. The number of tracking scans of which each dataset is comprised is shown in brackets.

*Frequency (%) of Occurrence*								
		*Euclidean Distance Between CT and Ultrasound Shifts*						
*Data Set (# of scans)*	≥5 mm	≥10 mm	≥15 mm	≥20 mm	≥25 mm	≥30 mm	≥40 mm	≥50 mm
General User (272)	88.8	42	17.9	9.4	4.9	2.7	1.3	0.9
Dedicated User (210)	81.9	35.7	9.5	2.9	1.0	0.5		
Expert User (153)	84.8	27.7	0.9					

**Table 2:** Frequency (%) at which discrepancies between first and second ultrasound tracking scans are equal to, or greater than, specific Euclidean distances. The number of tracking scans of which each dataset is comprised is shown in brackets.

*Frequency (%) of Occurrence*					
	*Euclidean Distance Between First and Second Ultrasound Localizations*				
*Data Set (# of scans)*	≥ 5 mm	≥ 10 mm	≥ 15 mm	≥ 20 mm	≥ 25 mm
General User (272)	88.4	38.4	17.9	5.4	3.6
Dedicated User (210)	68	12	3	2	
Expert User (153)	64.6	8.3			

## V. CONCLUSIONS

Results obtained with a pool of trained general users performing both scan acquisition and PRV delineation (as promoted by the manufacturer) resulted in poor correlation between CT and Ultrasound prostate shifts. The mean discrepancy between CT and ultrasound was greater than 10 mm, with a discord of 5 mm or more occurring for almost 90% of all tracking scans. Used in this manner, the Clarity system fails to yield the accuracy required for modern radiotherapy purposes.

Retrospective analysis of the original general user dataset by both a dedicated user and Resonant Medical expert user resulted in significant reductions in the discrepancy observed between paired ultrasound localizations. Despite this, the occurrence rates of disparity of greater than 5 mm between CT and ultrasound failed to drop below 80%. Even with a culling of over 56% of the original data, this ultrasound system fails to deliver an acceptable level of geometric accuracy with regard to prostate localization. This excessive cull rate argues strongly against the manufacturer's recommended use of the Clarity system by general users.

The improvements in outcome obtained with the dedicated user and the expert review provided by a Resonant Medical strongly suggest that the use of dedicated expert users should be investigated in regard to ultrasound scan acquisition. Unfortunately, this was beyond the scope of this investigation. The persistent discord between CT and ultrasound prostate localization, irrespective of the level of expertise applied to the analysis of the original data, indicates the existence of a fundamental inequity in the localization capabilities of these two modalities. These findings are in keeping with the study of Johnston et al.^(^ ^15^ ^)^ who concluded that this ultrasound system was incapable of safely replacing fiducial markers for daily prostate radiotherapy alignment, and suggested user variability as a root cause of the unacceptable variances in ultrasound localization that they observed.
